# The Ghost Map

**DOI:** 10.3201/eid1307.070357

**Published:** 2007-07

**Authors:** Ralph R. Frerichs

**Affiliations:** * University of California at Los Angeles, Los Angeles, California, USA

Widely viewed as the “father of contemporary epidemiology,” Dr. John Snow is among the most famous of public health figures (*1*). His “grand experiment” in 1854 (comparing cholera deaths in South London households that had consumed contaminated water with those that had not consumed contaminated water) is often considered a classic (*2*), but the Broad Street pump outbreak is perhaps the more famous historical account and is the subject of Steven Johnson’s new book, The Ghost Map*.*Dr. Snow wrote: “The most terrible outbreak of cholera which ever occurred in this kingdom, is probably that which took place in Broad Street, Golden Square, and the adjoining streets, a few weeks ago. Within two hundred and fifty yards of the spot where Cambridge Street joins Broad Street, there were upwards of five hundred fatal attacks of cholera in ten days. The mortality in this limited area probably equals any that was ever caused in this country, even by the plague; and it was much more sudden, as the greater number of cases terminated in a few hours” (*2*).Although this 1854 outbreak is mentioned in many public health and epidemiology texts, the focus is usually on data gathering and presentation, and the actions taken to address the outbreak. What is not often conveyed is the social environment of the times or the role of Reverend Henry Whitehead in dealing with this fearsome outbreak. Steven Johnson addresses these omissions in The Ghost Map and brings forth aspects of John Snow’s life in an insightful, riveting manner.Johnson’s opening sentences provide a sense of what is to come: “It is August 1854, and London is a city of scavengers. Just the names alone read now like some kind of exotic zoological catalogue: bone pickers, rag-gatherers, pure-finders, dredgermen, mud-larks, sewer-hunters, dustmen, night-soil men, bunters, toshers and shoremen.” He goes on to describe their roles in Victorian London and provides the reader with an intimate feel for local life, notably the travails of getting water and disposing of sewage. Along the way, the reader meets a local clergyman, Henry Whitehead, whose affable nature is in contrast to that of the more stoic John Snow. Yet, these 2 men of varied backgrounds become entwined by the Broad Street outbreak, using their complementary skills to help solve an epidemiologic mystery.The Ghost Map scarcely mentions the contributions of William Farr and other notables of the times. Instead, being a novel rather than a treatise, the book attempts to breathe life into a few seminal characters. Johnson is an excellent writer. His words evoke strong images that revolve in the mind. He uses London and Snow’s classic map of the 1854 outbreak as the focal points of his story, along with the removal of the Broad Street pump handle and the discovery of the probable index case. This is a good read, highly recommended for those open to the contributions of our forebearers in public health and the link of 19th-century London to modern day urban life.
